# Supplementary material for: In Patients with Severe COVID-19, the Profound Decrease in the Peripheral Blood T-Cell Subsets Is Correlated with an Increase of QuantiFERON-TB Gold Plus Indeterminate Rates and Reflecting a Reduced Interferon-Gamma Production
Source: Life (Basel). 2022 Feb 7;12(2):244. doi: 10.3390/life12020244 (PMC8880410; doi:10.3390/life12020244)
Supplement: Supplementary file 1 [file life-12-00244-s001.zip › life-1544950-supplementary.pdf]

In patients with severe COVID-19, the profound decrease in the peripheral blood T-cell subsets is correlated with an increase of QuantiFERON-TB Gold Plus indeterminate rates and reflecting a reduced interferon-gamma production

Alessandra Imeneo[1,†], Grazia Alessio[1,†], Andrea Di Lorenzo[1], Laura Campogiani[2], Alessandra Lodi[1], Filippo Barreca[1], Marta Zordan[1], Virginia Barchi[1], Barbara Massa[1], Simona Tedde[1], Angela Crea[1], Pietro Vitale[2], Ilaria Spalliera[2], , Mirko Compagno[2], Luigi Coppola[2], Luca Dori[2], Vincenzo Malagnino[1,2], Elisabetta Teti[2], Massimo Andreoni[1,2], Loredana Sarmati[1,2, †] and Marco Iannetta[1,2,*,†]

[1] Department of System Medicine, University of Rome Tor Vergata, Via Montpellier 1, 00133 Rome, Italy.
[2] Infectious Disease Clinic, Policlinico Tor Vergata, Viale Oxford 81, 00133 Rome, Italy.
[*] Correspondence: marco.iannetta@uniroma2.it
[†] These authors contributed equally to this work

Supplementary material

Asymptomatic patients (n=20) were removed from the analysis and the results are shown in Supplementary Tables S1-S5. Total subject included in the analysis = 400 COVID-19 symptomatic subjects.

Supplemntary Table S1. Demographics and clinical characteristic of the cohort, overall and after stratification for outcome (survivors vs non-survivors) and COVID-19 severity (non-severe vs severe).

	All patients (N=400)	Survivors (77.5%)	Non-survivors (22.5%)	p*	Non-severe (49.8%)	Severe (50.3%)	p**
Male/Female (%)	66.0/34.0	63.2/36.8	75.6/24.4	0.030	58.3/41.7	73.6/26.4	0.001
Age (years)	65.0 (52.0-76.0)	61.0 (49.0-72.0)	73.0 (68.3-82.0)	<0.001	59.0 (47.5-75.0)	69.0 (57.0-77.0)	<0.001
Age > 65 years (%)	48.9	39.4	81.1	<0.001	38.2	59.2	<0.001
Charlson Index	4.0 (2.0-5.0)	3.0 (2.0-5.0)	5.5 (4.0-7.0)	<0.001	3.0 (1.0-5.0)	4.0 (3.0-6.0)	<0.001
Cardiovascular (%)	55.8	50.3	74.4	<0.001	49.2	62.2	0.009
Diabetes (%)	22.8	20.0	32.2	0.015	21.6	23.9	0.588
Obesity (%)	20.3	21.0	17.8	0.507	17.1	23.4	0.117
Pulmonary (%)	13.5	9.7	26.7	<0.001	10.1	16.9	0.045
Neurological/ Psychiatric (%)	13.3	10.0%	24.4	<0.001	12.6	13.9	0.687
Solid tumor (%)	13.3	11.0	21.1	0.012	10.1	16.4	0.060
Endocrinological (%)	10.0	10.7	7.8	0.425	10.6	9.5	0.714
Renal (%)	9.0	4.8	23.3	<0.001	8.0	10.0	0.505
Cerebrovascular (%)	7.3	5.2	14.4	<0.003	6.0	8.5	0.349
Hematological (%)	6.3	4.2	13.3	0.002	2.5	9.9	0.002
Immunological/ Rheumatological (%)	5.5	6.1	3.3	0.306	8.5	2.5	0.008
Viral Hepatitis (%)	2.0	1.9	2.2	0.864	2.0	2.0	0.989
Other comorbidities (%)	24.0	22.9	27.8	0.340	22.6	25.4	0.518
ICU (%)	20.3	8.7	60.0	<0.001	3.0	37.3	<0.001
Steroid treatment (%)	63.3	57.1	84.4	<0.001	35.2	91.0	<0.001
Anti-IL6R (%)	10.2	10.0	11.1	0.760	5.0	15.4	<0.001
ΔT Symp-NPhS (days)	5.0 (1.0-8.0)	5.0 (2.0-8.0)	4.0 (0.0-8.0)	0.102	4.0 (1.0-7.0)	5.0 (2.0-8.0)	0.237
ΔT NPhS-QFTP (days)	4.0 (2.0-7.0)	4.0 (2.0-7.0)	3.0 (2.0-7.8)	0.410	4.0 (2.0-7.0)	4.0 (2.0-8.0)	0.905

ΔT Symp-QFTP (days)	10.0 (7.0-14.0)	10.0 (7.0-14.0)	9.0 (5.0-12.8)	0.051	10.0 (6.0-14.0)	10.0 (7.0-14.0)	0.479
ΔT Symp-TBNK (days)	8.5 (5.0-12.0)	9.0 (5.0-12.0)	7.0 (4.0-11.0)	0.036	8.0 (4.0-11.0)	9.0 (5.0-12.0)	0.045
ΔT TBNK-QFTP (days)	0.0 (0.0-3.0)	0.0 (0.0-3.0)	0.0 (0.0-3.0)	0.993	0.0 (0.0-3.0)	0.0 (0.0-3.0)	0.013

Quantitative variables are presented as median (interquartile range); categorical variables are presented as percentages.

*comparison between survivors vs non-survivors; **comparison between non-severe vs severe COVID-19 patients.

Cardiovascular comorbidities included heart failure, coronary artery disease, cardiomyopathies and hypertension; diabetes in-cluded both type I and II diabetes mellitus; obesity was defined as a body mass index ⩾30 kg/m2; pulmonary comorbidities in-cluded all kind of chronic lung diseases; neurological/psychiatric comorbidities consisted of all chronic neurological conditions, including dementia, as well as mental health disorders and depression; solid tumor included all malignant neoplastic diseases; endocrinological comorbidities included non-neoplastic endocrinological disorders; renal comorbidities included chronic kid-ney disease; cerebrovascular comorbidities included stenosis, thrombosis, embolism and hemorrhages; hematological comor-bidities included malignancies, red blood cell disorders, platelet disorders; immunological/rheumatological disorders included autoimmune and connective tissue diseases; viral hepatitis included active or past HBV and/or HCV infection; other comorbidi-ties included clinical relevant conditions not included in the above mentioned conditions. ICU: Intensive care unit; anti-IL6R: anti-interleukine-6 receptor; ΔT Symp-NPhS: days from symptoms' onset to the first positive nasopharyngeal swab for SARS-CoV-2 RNA detection with RT-PCR; ΔT NPhS-QFTP: days from the first positive nasopharyngeal swab for SARS-CoV-2 RNA detection with RT-PCR to QuantiFERON-TB Gold Plus sampling day; ΔT Symp-QFTP: days from symptoms' onset to Quan-tiFERON-TB Gold Plus sampling day; ΔT Symp-TBNK: days from symptoms' onset to peripheral blood T-, B-, NK-lymphocyte assessment day; ΔT TBNK-QFTP: days from peripheral blood T-, B-, NK-lymphocyte assessment to QuantiFERON-TB Gold Plus sampling day.

Supplementary Table S2. Laboratory parameters at Infectious diseases ward admission of the study cohort, overall and after stratifi-cation for outcome (survivors vs non-survivors) and COVID-19 severity (non-severe vs severe).

	All patients	Survivors	Non-survivors	p*	Non-severe	Severe	p**
Neutrophils (cells/µl)	4765 (3170-7553)	4505 (2910-6690)	7010 (3857-9408)	<0.001	3840 (2480-5715)	6430 (3958-8997)	<0.001
Lymphocytes (cells/µl)	990 (628-1410)	1110 (730-1505)	630 (407-855)	<0.001	1250 (907-1680)	740 (497-1022)	<0.001
N/L ratio	5.1 (2.3-10.0)	4.2 (2.0-7.9)	11.6 (5.4-17.9)	<0.001	3.2 (1.6-5.2)	8.4 (5.0-14.6)	<0.001
CRP (mg/l)	62.8 (27.1-114.5)	44.8 (21.3-98.8)	113.8 (70.3-151.4)	<0.001	37.5 (13.5-77.3)	87.5 (45.4-139.4)	<0.001
IL-6 (pg/ml)	17.4 (7.6-44.1)	13.9 (6.2-32.9)	41.1 (16.8-67.5)	<0.001	13.4 (5.7-31.7)	21.1 (11.3-48.9)	<0.001
TNF-α (pg/ml)	12.9 (7.2-26.6)	12.7 (7.2-26.8)	14.1 (7.2-24.2)	0.919	16.2 (9.8-33.0)	11.1 (6.6-19.1)	<0.001
D-dimer (ng/ml)	904 (511-1583)	804.0 (480.5-1471.3)	1317.0 (933.5-2235.0)	<0.001	788.6 (437.0-13630)	1078.0 (654.0-1878.0)	<0.001
Fibrinogen (mg/dl)	551 (429-687)	533.0 (413.5-679.0)	624.0 (461.5-733.5)	0.012	478.0 (371.5-627.0)	625.0 (501.5-736.0)	<0.001
Ferritin (mg/dl)	635 (284-1213)	528.0 (239.0-1064.0)	1007.0 (684.0-1791.0)	<0.001	357.0 (178.3-923.5)	888.5 (435.5-1773.3)	<0.001

Quantitative variables are presented as median (interquartile range).

*comparison between survivors vs non-survivors; **comparison between non-severe vs severe COVID-19 patients.

Reference values: CRP (mg/l): 0–5.00; IL-6 (pg/ml): <50; TNF-alpha (pg/ml): <12.4; D-dimers (ng/ml): 0–500.00; Fi-brinogen (mg/dl): 200.00–400.00; Ferritin: 4.63-204.00. N/L ratio: neutrophils-to-lymphocytes ratio; CRP: C-reactive protein; IL-6: interleukine-6, TNF-α: tumor necrosis factor-α.

Supplementary Table S3. QFT-Plus results of the study cohort, overall and after stratification for outcome (survivors vs non-survivors) and COVID-19 severity (non-severe vs severe).

	**All patients (N=400)**	**Survivors (N=310)**	**Non-survivors (N=90)**	**p***	**Non-severe (N=213)**	**Severe (N=207)**	**p****
Pos/Neg/Indet (%)	8.0/69.5/22.5	9.0/71.6/19.4	4.4/62.2/33.3%	0.012	9.5/79.9/10.6	6.5/59.2/34.3	<0.001
Det/Indet (%)	77.5/ 22.5	80.7/19.4	66.7/33.3	0.005	89.4/10.6	65.7/34.3	<0.001
TB1 (IU/ml)	0.106 (0.055-0.257)	0.114 (0.062-0.304)	0.080 (0.042-0.163)	0.003	0.123 (0.064-0.325)	0.99 (0.049-0.1902)	0.013
TB1-Nil (IU/ml)	0.005 (0.000-0.042)	0.007 (0.000-0.051)	0.003 (0.000-0.019)	0.038	0.007 (0.000-0.046)	0.005 (0.000-0.032)	0.106
TB2 (IU/ml)	0.105 (0.058-0.248)	0.111 (0.066-0.271)	0.081 (0.044-0.168)	<0.001	0.113 (0.069-0.294)	0.093 (0.049-0.196)	0.002
TB2-Nil (IU/ml)	0.005 (0.000-0.042)	0.007 (0.000-0.051)	0.001 (0.000-0.030)	0.044	0.007 (0.000-0.046)	0.003 (0.000-0.039)	0.110
Mitogen (IU/ml)	3.830 (0.938-10.000)	5.775 (1.258-10.000)	1.390 (0.458-3.310)	<0.001	9.740 (2.120-10.000)	1.800 (0.430-6.110)	<0.001
Mitogen-Nil (IU/ml)	3.433 (0.641-9.631)	5.470 (1.019-9.784)	1.212 (0.259-2.858)	<0.001	8.380 (1.780-9.881)	1.366 (0.255-5.967)	<0.001

Quantitative variables are represented as median (interquartile range); categorical variables are presented as percentages.

*comparison between survivors vs non-survivors; **comparison between non-severe vs severe COVID-19 patients.

QFT-Plus: QuantiFERON-TB Gold Plus; Pos: positive; Neg: negative; Indet: indeterminate; Det: determinate.

Supplementary Table S4. Demographic, clinical and laboratory parameters in COVID-19 patients with a deter-minate or indeterminate result at the QFT-Plus assay.

	**All patients**	**QFT-Plus determinate**	**QFT-Plus indeterminate**	**p**
Male/Female (%)	66.0/34.0	63.5/36.5	74.4/25.6	0.055
Age (years)	65.0 (52.0-76.0)	63.5 (50-76)	70 (57-78.6)	0.007
Age > 65 years (%)	48.9	45.5	60.0	0.015
Charlson Index	4.0 (2.0-5.0)	4.0 (2.0-5.0)	4.0 (3.0-6.0)	0.022
ΔT NPhS-QFTP (days)	4.0 (2.0-7.0)	4 (2-7)	5 (2-8)	0.190
ΔT Symp-QFTP (days)	10.0 (7.0-14.0)	10 (6-13)	11 (8-15)	0.086
ΔT TBNK-QFTP (days)	0.0 (0.0-3.0)	0 (0-3)	0 (0-3)	0.604
ICU (%)	20.3	17.1%	31.1	0.004
Neutrophils (cells/µl)	4765 (3170-7553)	4275 (2835-6750)	6675 (5363-9978)	<0.001
Lymphocytes (cells/µl)	990 (628-1410)	1075 (690-1515)	680 (448-1000)	<0.001
N/L ratio	5.1 (2.3-10.0)	4.0 (2.0-8.0)	10.1 (5.8-18.6)	<0.001
CRP (mg/l)	62.8 (27.1-114.5)	53.1 (23.9-107.7)	101.5 (44.0-148.2)	<0.001
IL-6 (pg/ml)	17.4 (7.6-44.1)	16.9 (7.5-43.4)	18.2 (9.8-47.5)	0.389
TNF-alpha (pg/ml)	12.9 (7.2-26.6)	14.4 (8.2-28.1)	9.9 (5.8-17.3)	<0.001
D-dimer (ng/ml)	904 (511-1583)	824.0 (480.5-1488.0)	1155.0 (730.0-2042.5)	<0.001
Fibrinogen (mg/dl)	551 (429-687)	533.0 (412.0-669.8)	631.0 (488.3-728.0)	0.003
Ferritin (mg/dl)	635 (284-1213)	481.8 (231.0-1021.0)	1096.0 (720.0-1839.0)	<0.001
# CD3+ (cells/µl)	633.5 (386.8-1030.5)	730.0 (453.0-1131.0)	382.0 (243.0-613.0)	<0.001
# CD3+CD4+ (cells/µl)	386.0 (232.8-636.0)	440.0 (274.0-712.0)	248.0 (152.0-446.0)	<0.001
# CD3+CD8+ (cells/µl)	195.0 (116.8-360.0)	240.0 (141.0-406.0)	107.0 (75.0-172.0)	<0.001
CD4/CD8 ratio	1.9 (1.2-2.8)	1.8 (1.2-2.5)	2.5 (1.7-3.6)	<0.001
# CD19+ (cells/µl)	106.5 (62.0-161.3)	107.0 (62.0-161.0)	106.0 (62.5-178.0)	<0.001
# CD3negCD16+CD56+ (cells/µl)	129.0 (79.8-216.3)	147.0 (90.0-227.0)	93.0 (57.0-160.0)	0.369

Quantitative variables are presented as median (interquartile range); qualitative variables are presented as percentages. Reference values: CRP (mg/l): 0–5.00; IL-6 (pg/ml): <50; TNF-alpha (pg/ml): <12.4; D-dimers (ng/ml): 0–500.00; Fibrinogen (mg/dl): 200.00–400.00; Ferritin: 4.63-204.00, CD3+(%): 55–84; CD3+(cells/µl): 690–2540; CD3+CD4+(cells/µl): 410–1590; CD3+CD8+(cells/µl): 190–1140; CD19+(cells/µl): 90–660; CD3negCD16+CD56+(cells/µl): 90–590; CD4/CD8 ratio: 1.5–2.5. QFT-Plus: QuantiFERON-TB Gold Plus; ΔT NPhS-QFTP: days from the first positive nasopharyngeal swab for SARS-CoV-2 RNA detection with RT-PCR to Quanti-FERON-TB Gold Plus sampling day; ΔT Symp-QFTP: days from symptoms' onset to Quanti-FERON-TB Gold Plus sampling day; ΔT TBNK-QFTP: days from peripheral blood T-, B-, NK-lymphocyte assessment to QuantiFERON-TB Gold Plus sampling day; N/L ratio: neutro-phils-to-lymphocytes ratio; CRP: C-reactive protein; IL-6: interleukine-6, TNF-α: tumor necrosis factor-α; #: absolute count.

Supplementary Table S5. Univariable and multivariable logistic regression analysis: factors associated with an indeterminate response of the QTF-Plus assay in COVID-19 patients.

	Univariable				Multivariable			
	**Odds ratio**	**95% CI**		**p**	**Odds ratio**	**95% CI**		p
Sex (M)	1.671	1.986	2.831	0.056	/	/	/	/
Age > 65 years	1.798	1.115	2.898	**0.016**	0.771	0.351	1.694	0.518
Charlson Index	1.110	1.012	1.217	**0.026**	1.146	0.928	1.332	0.252
Tocilizumab	1.386	0.617	3.114	0.429	/	/	/	/
Sarilumab	1.747	0.428	7.129	0.605	/	/	/	/
Anti-IL-6R before QFT-Plus	2.561	0.946	6.936	0.064	/	/	/	/
Steroid before QFT-Plus	0.390	0.088	1.722	0.214	/	/	/	/
N/L Ratio	1.099	1.065	1.135	**<0.001**	1.024	0.980	1.071	0.291
CRP	1.006	1.003	1.009	**<0.001**	0.998	0.992	1.005	0.564
IL-6	1.000	0.999	1.001	0.791	/	/	/	/
D-dimers	1.000	1.000	1.000	0.068	/	/	/	/
Fibrinogen	1.002	1.001	1.003	**0.004**	1.000	0.997	1.002	0.865
Ferritin	1.000	1.000	1.000	**0.002**	1.000	1.000	1.000	0.212
# CD3+	0.997	0.996	0.998	**<0.001**	0.998	0.996	0.999	**0.002**
CD4/CD8 ratio	1.309	1.110	1.543	**0.001**	1.622	1.266	2.077	**<0.001**
# CD19+	1.000	0.999	1.000	0.666	/	/	/	/
# CD3negCD16+CD56+	0.996	0.994	0.999	**0.003**	1.000	0.997	1.003	0.985

QFT-Plus: QuantiFERON-TB Gold Plus; 95% CI: 95% confidence interval; N/L ratio: neutro-phils-to-lymphocytes ratio; CRP: C-reactive protein; IL-6: interleukine-6; #: absolute counts.
